# Isolation and Characterization of Nitrate-Reducing Bacteria as Potential Probiotics for Oral and Systemic Health

**DOI:** 10.3389/fmicb.2020.555465

**Published:** 2020-09-15

**Authors:** Bob T. Rosier, Eva M. Moya-Gonzalvez, Paula Corell-Escuin, Alex Mira

**Affiliations:** Department of Health and Genomics, Center for Advanced Research in Public Health, FISABIO Foundation, Valencia, Spain

**Keywords:** nitrate reduction, probiotics, pH buffering capacity, caries, oral microbiota, nitric oxide, cardiovascular diseases, *Rothia*

## Abstract

Recent evidence indicates that the reduction of salivary nitrate by oral bacteria can contribute to prevent oral diseases, as well as increase systemic nitric oxide levels that can improve conditions such as hypertension and diabetes. The objective of the current manuscript was to isolate nitrate-reducing bacteria from the oral cavity of healthy donors and test their *in vitro* probiotic potential to increase the nitrate-reduction capacity (NRC) of oral communities. Sixty-two isolates were obtained from five different donors of which 53 were confirmed to be nitrate-reducers. Ten isolates were selected based on high NRC as well as high growth rates and low acidogenicity, all being *Rothia* species. The genomes of these ten isolates confirmed the presence of nitrate- and nitrite reductase genes, as well as lactate utilization genes, and the absence of antimicrobial resistance, mobile genetic elements and virulence genes. The pH at which most nitrate was reduced differed between strains. However, acidic pH 6 always stimulated the reduction of nitrite compared to neutral pH 7 or slightly alkaline pH 7.5 (*p* < 0.01). We tested the effect of six out of 10 isolates on *in vitro* oral biofilm development in the presence or absence of 6.5 mM nitrate. The integration of the isolates into *in vitro* communities was confirmed by Illumina sequencing. The NRC of the bacterial communities increased when adding the isolates compared to controls without isolates (*p* < 0.05). When adding nitrate (prebiotic treatment) or isolates in combination with nitrate (symbiotic treatment), a smaller decrease in pH derived from sugar metabolism was observed (*p* < 0.05), which for some symbiotic combinations appeared to be due to lactate consumption. Interestingly, there was a strong correlation between the NRC of oral communities and ammonia production even in the absence of nitrate (*R* = 0.814, *p* < 0.01), which indicates that bacteria involved in these processes are related. As observed in our study, individuals differ in their NRC. Thus, some may have direct benefits from nitrate as a prebiotic as their microbiota naturally reduces significant amounts, while others may benefit more from a symbiotic combination (nitrate + nitrate-reducing probiotic). Future clinical studies should test the effects of these treatments on oral and systemic health.

## Introduction

The salivary glands contain electrogenic sialin 2NO_3_^–^/H^+^ transporters to concentrate plasma nitrate into the saliva (Qu et al., 2016). This leads to salivary nitrate concentrations that are around ten times higher than plasma during fasting (100–500 μM compared to 10–50 μM), which can go up to 5–8 mM after a nitrate-containing meal [reviewed by Lundberg and Govoni (2004) and Hezel and Weitzberg (2015)]. Foods that naturally contain significant amounts of nitrate are fruits and vegetables, which are both unequivocally associated with health benefits. It is estimated that we obtain more than 80% of nitrate from vegetables (Lundberg et al., 2018).

Nitrate-reducing oral bacteria, including representatives of *Neisseria*, *Rothia*, *Veillonella*, *Actinomyces*, *Corynebacterium*, *Haemophilus*, and *Kingella* reduce nitrate to nitrite (Grant and Payne, 1981; Doel et al., 2005; Hyde et al., 2014). Human cells cannot reduce nitrate, but there are several enzymatic and non-enzymatic processes that convert nitrite into nitric oxide (Hezel and Weitzberg, 2015). For example, in the acidic gastric juice, nitrite is decomposed to nitrogen oxides, such as nitric oxide (NO), which is essential for the antimicrobial activity of the stomach (Lundberg and Govoni, 2004). It should be noted that anti-oxidants and polyphenols in vegetables and fruits prevent the formation of carcinogenic *N*-nitroso compounds from nitrite, while stimulating nitric oxide production (Ward, 2009; Kobayashi et al., 2015).

In the blood vessels, nitrite reacts with hemoglobin to form nitric oxide, which apart from being antimicrobial, is also an important vasodilator of the human body (Hezel and Weitzberg, 2015). It was shown that an antiseptic mouthwash acutely increases blood pressure by disrupting nitrate reduction by oral bacteria (Kapil et al., 2013). Nitrate-rich supplements, in turn, stimulate nitrate reduction by the oral microbiota resulting in a lowering of blood pressure (Vanhatalo et al., 2018). This pathway (i.e., the nitrate–nitrite–nitric oxide pathway) can also increase sport performance and has apparent antidiabetic effects (Lundberg et al., 2018). In light of this, antiseptic mouthwash has shown to interfere with post-exercise hypotension (Cutler et al., 2019) and over-the-counter mouthwash correlated with diabetes and pre-diabetes development (Joshipura et al., 2017).

Nitrate in the form of lettuce juice has also been shown to reduce gingival inflammation compared to a placebo (nitrate-depleted lettuce juice) (Jockel-Schneider et al., 2016). Additionally, nitrate prevented acidification by oral bacteria (Li et al., 2007) and the nitrate reduction capacity (NRC) of the oral microbiota correlated negatively with caries abundance (Doel et al., 2004). Recently, Rosier et al. (2020) have proposed that nitrate reduction stimulates eubiosis (i.e., an increase in health-associated species and functions) of the oral microbiota (Rosier et al., 2020). Specifically, nitrate reduction prevented acidification and the resulting overgrowth of cariogenic bacteria by increasing lactate consumption and ammonia production. Additionally, nitrate increased health-associated nitrate-reducing genera, while decreasing strictly anaerobic periodontal diseases- and halitosis-associated bacteria, which could be sensitive to oxidative stress caused by nitric oxide. Nitrate reducing bacteria, such as representatives of *Neisseria*, *Rothia*, *Actinomyces*, and *Kingella* have been associated to oral health in many 16S rRNA sequencing studies and this could be related with their capacity to reduce nitrate (Mashimo et al., 2015; Rosier et al., 2018; Rosier et al., 2020). In conclusion, current data suggest that nitrate-reduction of the oral microbiota contributes to a healthy host physiology and appears to stimulate oral health.

The amount of nitrate-reducing species varies among individuals and, accordingly, the NRC as well (Liddle et al., 2019). Individuals with low baseline levels of nitrate-reducing species could use nitrate as a prebiotic to increase the levels of these bacteria over time. For example, after 1–4 weeks of beetroot consumption (a vegetable with high nitrate levels), the salivary levels of *Neisseria* and *Rothia* increased significantly (Velmurugan et al., 2016; Vanhatalo et al., 2018). Alternatively, in individuals lacking nitrate-reducing species, a direct increase could be achieved by the addition of nitrate-reducing probiotics. In a seminal study, Doel et al. (2005) isolated 99 oral bacteria that produced nitrite in the presence of nitrate under anaerobic conditions and 33 under aerobic conditions, but did not further test the effects of these isolates on oral communities. Apart from their ability to produce nitrite, nitric oxide or ammonia, oral probiotics should not be acidogenic (a feature associated with dental caries) and from a technological point of view should ideally be capable of fast aerobic growth to enable large-scale production (Lacroix and Yildirim, 2007).

The aim of our current study was therefore to isolate nitrate-reducing oral strains under aerobic conditions and make a selection of isolates that were most suitable from a biomedical and industrial point of view. To achieve this, a protocol was applied to obtain isolates with a high NRC, fast growth rate and low acidogenicity. Additionally, their genomes were sequenced and analyzed for functional predictions and for the detection of potentially harmful genes. To test our hypothesis that oral communities could benefit from nitrate-reducing isolates, six selected isolates were added to *in vitro* oral biofilms grown from saliva of different individuals that varied in NRC. The NRC was determined and changes in nitrate-related metabolism (e.g., ammonia and lactate production) monitored. Oral biofilm colonization was tested by 16S rRNA sequencing. All experimental data were used to assess the *in vitro* probiotic potential of nitrate-reducing oral bacteria as a first step to evaluate their possible use for oral and systemic health.

## Materials and Methods

### Donor Selection and Sample Procedure

For probiotic isolation, five young adults (1 male, 4 female, age 23–32) were selected with all teeth and good oral health, which was assessed by an experienced dentist. Individuals were excluded if they showed bleeding on probing or a periodontal pocket below 3 mm; a cavitated lesion or filling in any tooth surfaces. The absence of nitrate reduction capacity, as determined by lack of salivary nitrite, was as an exclusion criterion (the salivary nitrite and pH measured in the morning before breakfast for all donors are shown in Supplementary Table S1). All individuals had a healthy blood pressure (i.e., systolic between 90 and 120, and diastolic between 60 and 80), which was measured with an Automatic Blood Pressure Monitor Model M6 Comfort IT (OMRON Healthcare Europe B.V., Hoofddorp, Netherlands). Plaque and tongue coating samples were collected by the dentist following Simón-Soro et al. (2013) and resuspended in 1 mL of PBS (Simón-Soro et al., 2013).

For saliva donor selection, individuals were recruited at the FISABIO institute if they reported not to have active caries during their last dental visit, nor any history of periodontitis. Nine individuals (3 male, 6 female, age 23–45) were selected and asked to donate saliva, which was used for *in vitro* oral community growth with and without nitrate-reducing isolates. Unstimulated saliva was collected by drooling in a sterile tube in a quiet room in the morning, at least half an hour after eating or drinking. Donors were instructed to have a normal breakfast and abstain from oral hygiene before saliva collection.

The fresh unstimulated saliva was always directly used in the experiments or kept at 4°C for less than 1 h before usage. All donors signed an informed consent form prior to sample collection and the protocol was approved by the Ethical Committee of DGSP-FISABIO (Valencian Health Authority) with code 27-05-2016. This study was carried out according to the relevant guidelines and regulations of the Declaration of Helsinki.

### Isolation of Nitrate-Reducing Bacteria

**FIGURE 1:**
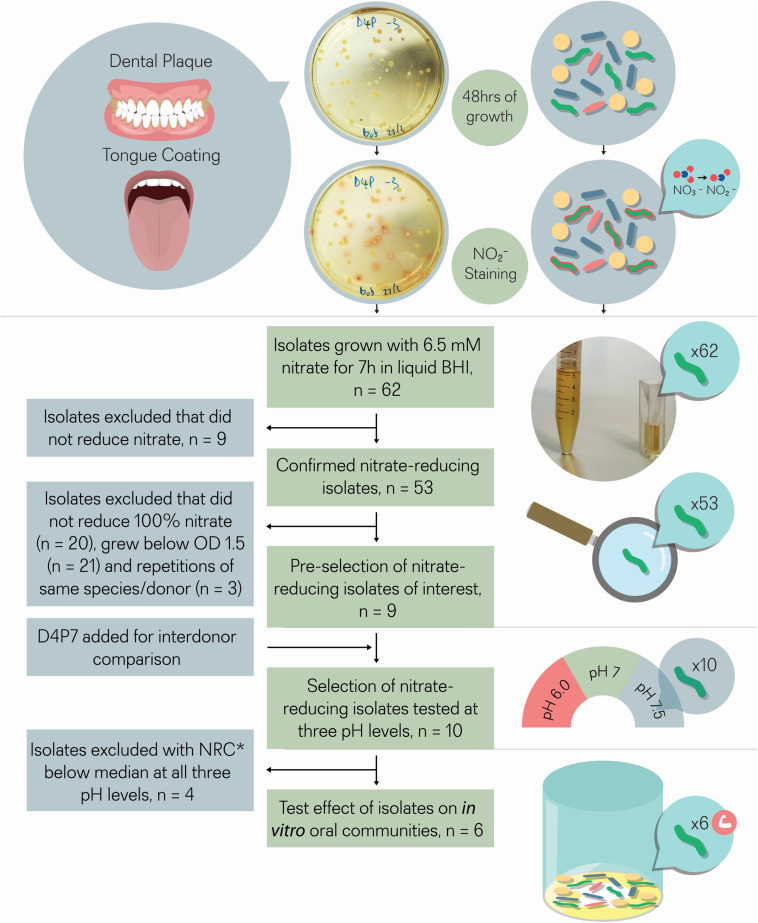
Isolate selection overview. Tongue coating and/or dental plaque samples were obtained from 5 different donors. Sixty-two colonies that produced a red color after adding Griess reagent, which stains nitrite, were isolated. The 62 isolates were incubated with 6.5 mM nitrate for 7 h for double confirmation. Nine out of 62 isolates did not reduce nitrate and were excluded, while the other 53 isolates did reduce (part of) the nitrate and were considered as confirmed cases. From these 53 isolates, nine isolates were selected that reduced all nitrate and grew well under aerobic conditions (OD > 1.5), a relevant feature for future large-scale production. One isolate (D4P7, *R. dentocariosa*) was added to compare nitrate reducing capacity with the three selected *R. dentocariosa* isolates between different donors. None of the 10 selected isolates acidified the pH of the glucose-containing medium (starting pH 7.3) below pH 6.8 after 7 h, which indicated that they were suitable probiotics from a caries point of view. The nitrate-reduction capacity of the final selection of 10 isolates was tested at three different pH levels (pH 6.5, pH 7, and pH 7.5) in buffered medium. The six isolates that reduced the nitrate-best at different pH levels were added to oral communities (from saliva) to test their effect on *in vitro* oral biofilm metabolism. *NRC, nitrate-reduction capacity.

Plaque or tongue samples in PBS were diluted 10^2^ to 10^7^ times and plated on Brain Heart Infusion (BHI) 1.4% agar plates (Merck Millipore, Burlington, MA, United States). Plates were incubated at 37°C under aerobic conditions during 2 days to obtain separated colonies in some of the dilutions. A protocol adapted from Doel et al. (2005) was employed to detect nitrate-reducing activity by individual colonies (Doel et al., 2005). This protocol consists of a double agar overlay method based on the Griess reaction that stains nitrite. Briefly, a plate with separated colonies grown from plaque or tongue samples was overlaid with 10 mL of 2.5% w/v agar with 1 mM sodium nitrate (NaNO_3_, Sigma-Aldrich, St. Louis, MO, United States) and incubated at 37°C for 10 min in which nitrate-reducing colonies would produce nitrite. Then, the first layer was overlaid with 10 mL 2.5% w/v agar containing the Griess reagents and incubated at room temperature for 10 min. Colonies with nitrate-reducing capability produced a red color due to the presence of nitrite (Figure 1). These colonies were then transferred to new BHI agar plates and incubated during 2 more days at 37°C. The nitrite-producing capability of the isolates was confirmed by repeating the double overlaid agar method for each isolate. Subsequently, one colony was passed to 5 mL of liquid BHI and incubated aerobically for 2 days at 37°C. After that, 0.5 ml of the medium was used to create a glycerol stock of each isolate for future experiments. The rest of the cells were resuspended in PBS and used for DNA extraction and sequencing.

### Nitrate Reduction Screenings of Bacterial Isolates

To evaluate the nitrate-related metabolism of the isolates, the concentrations of nitrate, nitrite, and ammonium, as well as pH levels, were measured in spent medium. Isolates were incubated in 5 mL BHI liquid medium overnight at 37°C. The next day, isolates were diluted in BHI to an OD of 0.01 and a final concentration of 6.5 mM nitrate, which is within the physiological range of salivary nitrate after a nitrate-containing meal [i.e., 5–8 mM (Hezel and Weitzberg, 2015)]. The tubes were then incubated for 7 h and 1 mL was taken at 4 and 7 h, after vortexing and, from this volume, 0.5 ml was used to measure the OD and 0.5 ml for the other measurements. A similar experiment was performed with 10 isolates selected as probiotic candidates, which were grown for 5 h in three types of buffered medium (100 mM MES, pH 6.0; 100 mM HEPES, pH 7.0; 100 mM HEPES pH 7.5, all Sigma-Aldrich) with 6.5 mM nitrate to keep a stable pH and evaluate the effect of different pH levels on the NRC of those isolates. All samples were frozen at −20°C before analysis of the supernatants.

### Effect of Isolates on *in vitro* Biofilms

Six out of 10 selected nitrate-reducing isolates were studied *in vitro* to define their effect when added to an oral microcosm community. These isolates were tested by growing them with saliva of nine different donors in 96-wells plates in which oral communities form biofilms on the bottom of wells (Mira et al., 2019). For each experiment, there were 4 conditions: control, nitrate (final concentration: 6.5 mM nitrate), control with isolate, and nitrate with isolate. For all samples, prepared in duplicate, 100 μl of BHI (with 0.05 mg/L haemin, 0.005 mg/L menadione and 0.2 mM vitamin K) were added to each well. Then, 100 μL of saliva (or BHI for negative controls) was added and, for the nitrate conditions, 10 μL of nitrate solution 162.5 mM was added (or 10 μL of BHI for control conditions). Before being added to the 96-well plate, the isolates were grown for 24 h. Then, 40 μL isolate in BHI solution with OD 1.5 was added (or 40 μL of BHI in conditions without isolates) to each well. The final concentration of nitrate was 6.5 mM and the starting OD of the isolate was 0.24. The 96-well plate was sealed to stimulate anaerobic conditions by preventing new oxygen from entering the wells, and incubated during 7 h at 37°C. After that, the supernatant was collected and stored at −20°C until measurements were performed. The remaining biofilms were resuspended in PBS for DNA isolation.

### DNA Isolation for Sequencing

DNA was extracted from pure cultures of the 53 confirmed nitrate-reducing isolates and also from the *in vitro* communities grown with isolates of two donors (D6 and D11). Pure cultures of isolates grown for 48 h in 4.5 ml BHI were centrifuged (15 min at 4,000 rpm) and the pellet resuspended in 100 μl PBS. After supernatant removal, duplicates of *in vitro* communities were resuspended together in 100 μl PBS and disaggregated for 30 s in a sonicator bath (model VCI-50, Raypa, Barcelona, Spain) at low ultrasound intensity. Total DNA was extracted with the MasterPure^TM^ Complete DNA and RNA Purification Kit (Epicentre Biotechnologies, Madison, WI, United States), following the manufacturer’s instructions, with the addition of lysozyme (Belda-Ferre et al., 2012). DNA was resuspended in 30 μl elution buffer and frozen at −20°C until further analysis.

### Taxonomic Classification of Nitrate-Reducing Isolates

For the taxonomic classification of the isolates, concentrations of DNA isolated from pure cultures were measured using a NanoDrop 1000 spectrophotometer (Thermo Scientific, Waltham, MA, United States). A PCR was performed to amplify the 16S rRNA gene of each isolate, using universal primers 8-F and 785-R for the 16S rRNA gene, comprising the hypervariable regions V1–V2–V3–V4. The PCR products were then purified using flat 96 well filter plates (NucleoFast 96 PCR; Macherey-Nagel, Düren, Germany) and sequenced at both ends by Sanger Technology at the Sequencing Unit of the University of Valencia (Valencia, Spain). To taxonomically assign the isolates, the sequences were compared by BLASTn (Altschul et al., 1997) against 16S ribosomal RNA sequences at NCBI nr database. Species assignment was confirmed by Average Nucleotide Identity (ANI) values using JSpeciesWS software (Richter et al., 2016).

### Genome Sequencing

Illumina libraries for all 10 selected isolates were generated using the Illumina XT Nextera library prep kit (catalog number FC-131-1024) starting from 0.2 ng/μl of purified gDNA measured by a Qubit double-stranded DNA (dsDNA) high-sensitivity assay kit (catalog number Q32851). Libraries were sequenced using a 2 × 150-bp paired-end run MiSeq reagent kit v2 (catalog number MS-102-2002) on an Illumina MiSeq sequencer.

Oxford Nanopore libraries were obtained from the same DNA extracted samples following the manufacturer’s standard protocol. Nanopore libraries were indexed and sequenced using type R9.4.1 (catalog number FLO-MIN106D) in a ONT MinION sequencer for 48 h. Both sequencing approaches were performed at the Sequencing Service of FISABIO-Public Health (Valencia, Spain).

Long ONT fast5 reads were base-called and transformed into fastq files by ONT Albacore Sequencing Pipeline Software (Pomerantz et al., 2018) and generated approximately four billion total bases in more than 850,000 reads (fastq-stats version 1.01^1^). These ONT long reads as well as the Illumina short reads were quality filtered and trimmed using Prinseq-lite (Schmieder and Edwards, 2011). Long ONT reads were assembled with Canu v1.8 using the nanopore preset parameters (Koren et al., 2017) into one single contig per genome. Their errors were corrected using Illumina quality-filtered and trimmed reads with Pilon software v1.23 (Walker et al., 2014). After three rounds of corrections, genomes were annotated by Prokka v1.13.3 (Seemann, 2014) and the downstream analysis was performed with these 10 annotated genomes.

### Genomic Analysis

In order to identify the presence of possible mobile genetic elements (MGEs), the annotated genomes were compared against the latest version of the ACLAME database (Leplae et al., 2010) where different kinds of MGEs are collected and classified at gene and protein levels. Sequences were compared by similarity using the program BLASTx (Camacho et al., 2009) identifying potential hits after filtering with the following criteria: minimum sequence identity higher than 80% of the gene length and coverage greater than 50%.

Potential antibiotic resistance genes (ARGs) were searched in the latest version of CARD database (McArthur et al., 2013). The detection of possible genes conferring pathogenicity was performed using the latest version of the virulence factors database (VFDB) which currently contains DNA sequences from 1,067 virulence factors from 951 bacterial strains having 32.252 virulence factor-related non-redundant genes information (Chen et al., 2016). Sequences were compared by similarity against both databases using the program BLASTn (Altschul et al., 1997), identifying potential hits with *E*-value < 10^–5^, sequence identity >80% and >50% sequence length as thresholds.

### Supernatant Analysis: Nitrate, Nitrite, Ammonium, Lactate, and pH Measurements

Nitrate, nitrite, lactate and pH were measured in supernatants with a Reflectoquant (Merck Millipore, Burlington, MA, United States) reflectometer. This method is based on the intensity of reflected light by two reactive pads on test trips that change in color intensity based on the concentration of a specific substance (Holden and Scholefield, 1995). The test strips (Reflectoquant, Merck Millipore) for pH had a range from pH 4–9, the strips for nitrate a range of 3–90 mg/l, the strips for nitrite a range of 0.5–25 mg/l and the strips for lactate a range of 3–60 mg/l. A method was used based on Helmke et al. (2009), and Ferrer et al. (2019) as described by Rosier et al. (2020). The concentration of ammonium in supernatants was measured spectrophotometrically by the Nessler Method (Santarpia et al., 2014). Accuracy of all procedures was confirmed by using standard solutions with known concentrations of the different compounds.

### Statistical Analysis

Statistical analysis was performed with SPSS 25 UK software (SPSS, Inc.). A Mann–Whitney *U* Test was applied to compare parameters between different (groups of) species. The Wilcoxon test was used to compare different conditions and the Spearman’s rank correlation coefficient was calculated for different parameters. Significant changes (*p* < 0.05) and trends (*p* < 0.1) were presented.

## Results

### Isolation of Nitrate-Reducing Bacteria and Testing Their Nitrate-Reduction Capacity

Tongue and dental plaque samples were plated from 5 different healthy donors (D1–D5). Potential nitrate-reducing colonies were detected by a red tone, produced by the Griess reaction resulting from nitrite production (Figure 1). In total, 33 nitrite-producing isolates were obtained from tongue (T) samples and 29 from dental plaque (P). Most isolates (74%) were obtained from two donors (D1 and D4, Supplementary Table S1).

**FIGURE 2:**
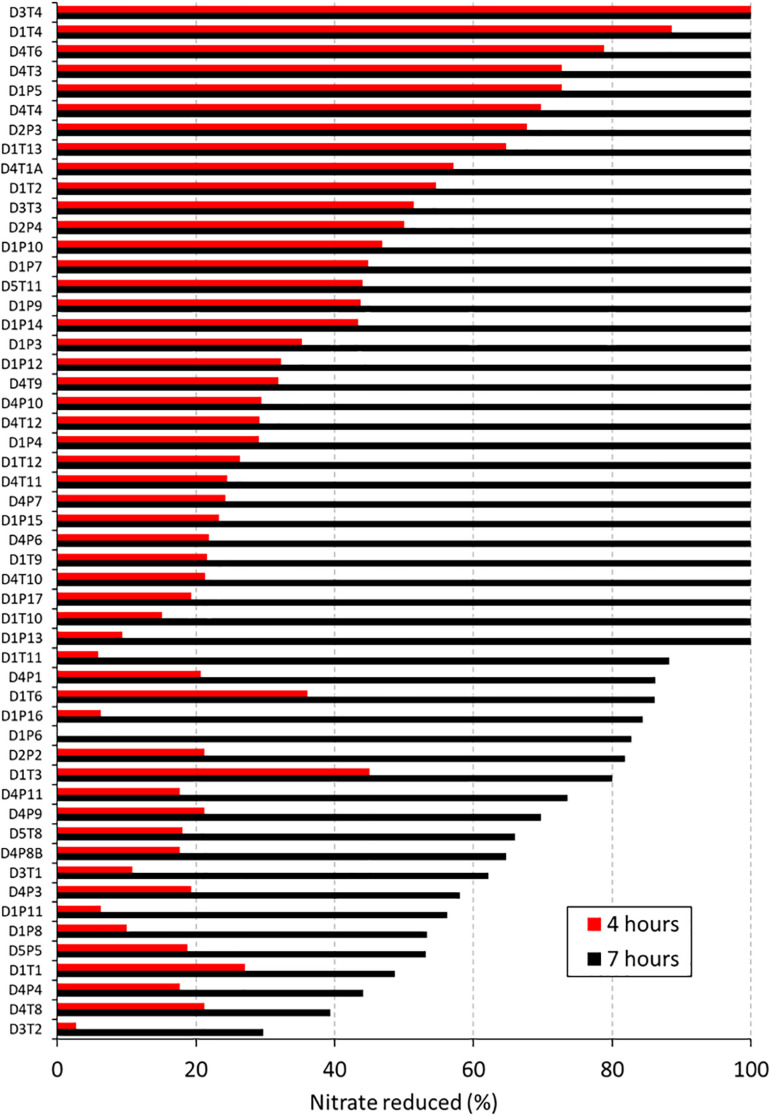
Nitrate reduction capacity of 53 isolates (1st screening). Bars show the percentage of nitrate reduced by 53 isolates after 4 h (red bars) and 7 (black bars) hours of incubation at 37°C under aerobic conditions with starting OD 0.01. Values represent the percentage of initially added nitrate (6.5 mM) that had been used up after 4 or 7 h of incubation. Codes of the 53 isolates are shown on the right, where D relates to the donor, T refers to tongue coating and P to dental plaque samples.

In an initial screening to quantify NRC, all 62 isolates were incubated with 6.5 mM nitrate during 4 and 7 h under aerobic conditions (Figure 2 and Table 1). Nine isolates, including strains of *Streptococcus salivarius*, *Streptococcus cristatus*, and *Streptococcus mitis*, did not reduce any of the nitrate and were excluded. The other 53 confirmed nitrate-reducing isolates were mostly classified as *Rothia* (i.e., 23 × *R. mucilaginosa*, 21 × *R. dentocariosa*, and 4 × *R. aeria*) and five as *Actinomyces* (3 × *Actinomyces viscosus* and 2 × *Actinomyces oris*, Table 1). All *R. mucilaginosa* isolates originated from the tongue, while all *R. dentocariosa* and *R. aeria* isolates were obtained from dental plaque (except for one *R. dentocariosa* isolate, D3T1, from the tongue). After 7 h, the average nitrite detected ranged from 1.20 to 10.39 mM (average 6.15 mM, *SD* 2.17 mM, Supplementary Table S2), suggesting that some bacteria produced (part of the) nitrite by other pathways than nitrate reduction.

**TABLE 1:** Confirmed nitrate-reducing isolates sorted by % nitrate reduced and optical density (OD) at 7 h.

Isolate	Species (16S BLAST)	Nitrate reduced (%)		OD		pH spent medium	
				
		4 h	7 h	4 h	7 h	4 h	7 h
D1P10*	*Rothia dentocariosa* ATCC17931	46.88	100.00	0.77	2.56	7.10	6.90
D3T4*	*Rothia mucilaginosa* DSM20746	100.00	100.00	1.00	2.16	7.10	6.80
D1P7*	*Rothia aeria* A1-17B	44.83	100.00	0.64	2.00	7.30	7.00
D4T4*	*Rothia mucilaginosa* DSM20746	69.70	100.00	0.65	1.97	7.20	6.80
D4T3	*Rothia mucilaginosa* DSM20746	72.73	100.00	0.69	1.96	7.20	6.70
D1P17*	*Rothia dentocariosa* ATCC17931	19.35	100.00	0.54	1.88	7.20	7.10
D4T6*	*Rothia mucilaginosa* DSM20746	78.79	100.00	0.79	1.87	7.20	6.80
D1P9	*Rothia dentocariosa* ATCC17931	43.75	100.00	0.57	1.76	7.20	7.20
D1P15*	*Rothia dentocariosa* ATCC17931	23.33	100.00	0.41	1.63	7.25	7.10
D4T9*	*Rothia mucilaginosa* DSM20746	31.91	100.00	0.57	1.60	7.20	7.10
D1P14	*Rothia aeria* A1-17B	43.33	100.00	0.48	1.56	7.30	7.10
D5T11*	*Rothia mucilaginosa* DSM20746	44.00	100.00	0.59	1.56	7.25	7.10
D1P12	*Rothia aeria* A1-17B	32.26	100.00	0.44	1.42	7.30	7.10
D1T4	*Rothia mucilaginosa* DSM20746	88.57	100.00	0.65	1.38	7.00	6.80
D1T13	*Rothia mucilaginosa* DSM20746	64.71	100.00	0.68	1.34	7.10	6.70
D4T12	*Rothia mucilaginosa* DSM20746	29.17	100.00	0.49	1.27	7.20	7.10
D1P13	*Rothia dentocariosa* ATCC17931	9.38	100.00	0.45	1.27	7.30	7.20
D1T9	*Rothia mucilaginosa* DSM20746	21.62	100.00	0.44	1.22	7.10	6.90
D1T12	*Rothia mucilaginosa* DSM20746	26.32	100.00	0.35	1.12	7.20	7.20
D4T10	*Rothia mucilaginosa* DSM20746	21.28	100.00	0.50	1.07	7.20	7.10
D4T1A	*Rothia mucilaginosa* DSM20746	57.14	100.00	0.44	1.04	7.20	7.00
D1T10	*Rothia mucilaginosa* DSM20746	15.15	100.00	0.29	1.03	7.30	7.10
D4T11	*Rothia mucilaginosa* DSM20746	24.49	100.00	0.41	0.96	7.30	7.10
D3T3	*Rothia mucilaginosa* DSM20746	51.35	100.00	0.33	0.85	7.20	7.00
D4P10	*Rothia dentocariosa* ATCC17931	29.41	100.00	0.35	0.81	7.10	7.00
D4P7*	*Rothia dentocariosa* ATCC17931	24.24	100.00	0.26	0.73	7.20	7.10
D4P6	*Rothia dentocariosa* ATCC17931	21.88	100.00	0.23	0.67	7.30	7.10
D2P4	*Rothia dentocariosa* ATCC17931	50.00	100.00	0.27	0.61	7.10	6.70
D1T2	*Rothia mucilaginosa* DSM20746	54.55	100.00	0.20	0.59	7.00	6.80
D1P3	*Actinomyces oris* ATCC27044	35.29	100.00	0.18	0.53	7.00	6.50
D2P3	*Rothia aeria* A1-17B	67.74	100.00	0.18	0.44	7.00	6.80
D1P5	*Rothia dentocariosa* ATCC17931	72.73	100.00	0.18	0.43	7.00	7.00
D1P4	*Rothia dentocariosa* ATCC17931	29.03	100.00	0.19	0.41	7.00	6.90
D1T11	*Rothia mucilaginosa* DSM20746	5.88	88.24	0.20	0.82	7.20	7.00
D4P1	*Rothia dentocariosa* ATCC17931	20.69	86.21	0.53	1.85	7.20	6.90
D1T6	*Rothia mucilaginosa* DSM20746	36.11	86.11	0.43	0.79	7.10	7.00
D1P16	*Rothia dentocariosa* ATCC17931	6.25	84.38	0.32	1.11	7.20	7.20
D1P6	*Rothia dentocariosa* ATCC17931	0.00	82.76	0.36	1.00	7.30	7.10
D2P2	*Rothia dentocariosa* ATCC17931	21.21	81.82	0.06	0.19	7.00	6.90
D1T3	*Rothia mucilaginosa* DSM20746	45.00	80.00	0.42	0.74	7.20	7.00
D4P11	*Rothia dentocariosa* ATCC17931	17.65	73.53	0.24	0.61	7.30	7.20
D4P9	*Rothia dentocariosa* ATCC17931	21.21	69.70	0.21	0.54	7.30	7.20
D5T8	*Rothia mucilaginosa* DSM20746	18.00	66.00	0.23	0.81	7.20	7.20
D4P8B	*Rothia dentocariosa* ATCC17931	17.65	64.71	0.24	0.45	7.20	7.20
D3T1	*Rothia dentocariosa* ATCC17931	10.81	62.16	0.25	0.48	7.20	7.10
D4P3	*Actinomyces viscosus* JCM8353	19.35	58.06	0.44	1.25	7.30	7.10
D1P11	*Rothia dentocariosa* ATCC17931	6.25	56.25	0.32	0.92	7.30	7.30
D1P8	*Rothia dentocariosa* ATCC17931	10.00	53.33	0.33	0.90	7.30	7.20
D5P5	*Actinomyces viscosus* JCM8353	18.75	53.13	0.24	0.57	7.20	7.10
D1T1	*Rothia mucilaginosa* DSM20746	27.03	48.65	0.29	0.50	7.20	7.10
D4P4	*Actinomyces viscosus* JCM8353	17.65	44.12	0.24	0.47	7.20	7.10
D4T8	*Actinomyces oris* JCM16131	21.21	39.39	0.24	0.49	7.20	7.10
D3T2	*Rothia mucilaginosa* DSM20746	2.70	29.73	0.18	0.23	7.20	7.10
Average (*SD*)	–	33.74 (23.11)	86.95 (19.92)	0.40 (0.19)	1.06 (0.56)	7.19 (0.10)	7.02 (0.17)

* Final selection of 10 nitrate-reducing isolates of interest.

After 4 h, the average nitrate reduced by the 53 isolates was 33.74% (*SD* 23.11%) and after 7 h this value increased to 86.95% (*SD* 19.92%). The starting OD of the medium was 0.01 and went up to an average OD of 0.40 (*SD* 0.19) after 4 h and OD 1.06 (*SD* 0.56) after 7 h. Isolates with acidogenic properties were excluded from the selection of potential probiotics, as acid production of isolates is associated with dental caries risk (Sanz et al., 2017). Only one nitrate-reducing isolate (i.e., D1P3 *A. oris*) acidified the pH of the glucose-containing BHI medium (starting pH 7.3) to pH 6.5 after 7 h of growth and the average of all isolates was pH 7.02 (*SD* 0.17), indicating there was little caries-associated acidification. With regard to this, one *S. salivarius* isolate, which was excluded because it did not reduce nitrate, lowered the pH to 5.5 under the same conditions.

**FIGURE 3:**
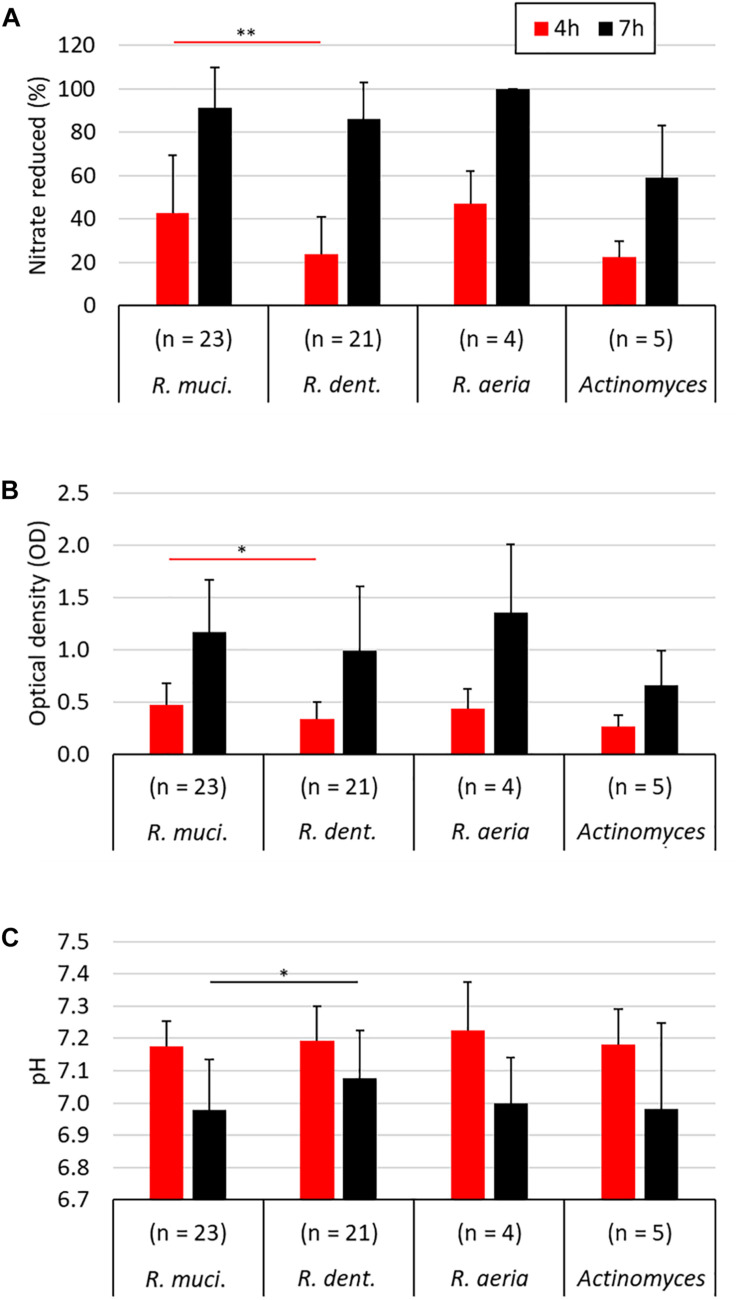
Comparison of different nitrate-reducing species isolated in the current work after 4 and 7 h of growth with 6.5 mM nitrate. The different *Rothia* species are grouped separately (23 *R. mucilaginosa* isolates, 21 *R. dentocariosa* isolates, and 4 *R. aeria* isolates) and all five *Actinomyces* isolates are represented together. Bars represent the nitrate reduced **(A)**, optical density **(B)**, and medium pH **(C)** after 4 and 7 h of growth. Only *R. mucilaginosa* (*R. muci*.) and *R. dentocariosa* (*R. dent*.), to which most isolates belonged, were statistically compared (Mann–Whitney *U* test). ^∗^*p* < 0.05, ^∗∗^*p* < 0.01.

When comparing all 23 *R. mucilaginosa* isolates and 21 *R. dentocariosa* isolates, *R. mucilaginosa* grew 1.39× more (*p* < 0.05, Figure 3B) and reduced 1.79× more nitrate (*p* < 0.01, Figure 3A) after 4 h. After 7 h there were no significant difference in OD and nitrate-reduction between the two species, but *R. mucilaginosa* had significantly decreased the pH by 0.1 points (*p* < 0.05, Figure 3C). This pattern of pH difference was consistent when comparing *R. mucilaginosa* and *R. dentocariosa* of donor 1 (*p* < 0.05) and donor 4 (*p* = 0.094, Supplementary Figure S1), which were the only two donors with enough isolates for an intra-donor comparison.

### Selection of Probiotic Candidates

Thirty-three isolates reduced 100% nitrate after 7 h (Table 1) and from these bacteria, 9 isolates were selected that grew to a final OD > 1.5 after 7 h (i.e., they grew well under aerobic conditions relevant for large-scale production, Table 1) for further analysis. These corresponded to three *R. dentocariosa* isolates from donor 1 (D1P10, D1P17, and D1P15) and three *R. mucilaginosa* isolates from donor 4 (D4T4, D4T6, and D4T9), which allowed to study strain differences. The other three isolates were *R. aeria* isolate D1P7 and *R. mucilaginosa* isolates D3T4 and D5T11. Additionally, an isolate from another donor (D4P7, *R. dentocariosa*) was added to the selection for inter-donor comparison of *R. dentocariosa* with the isolates of donor 1. These 10 selected isolates were further studied by whole genome sequencing, genome analysis and nitrate reduction quantification under different pH levels, and their effect on *in vitro* oral communities was tested.

### Genome Analysis and Identification of Possible Virulence Genes

After the whole genome sequencing procedure, sequences obtained by Illumina and Oxford Nanopore Technologies (ONT) procedures were quality-filtered, corrected and combined to produce a final assembly (Table 2). The genome sequences of all 10 isolates could be assembled into one single contig, except the sequences of D5T11 (Supplementary Table S3), which had less ONT sequences that passed the quality filter (only 8.73%).

**TABLE 2:** Whole genome sequence analysis, assembly information and species identification of the 10 selected isolates.

Isolate	Illumina sequences	ONT sequences	ONT mean length (bp)	Estimated genome size (bp)	Contigs	Mean coverage	Closest sequenced genome	ANI value*
D1P7	1.427.028	43.207	8.085	2.654.231	1	74×	*Rothia aeria* C6B	97,4
D1P10	1.649.834	34.991	9.775	2.486.471	1	93×	*Rothia dentocariosa* NCTC10917	96,58
D1P15A	195.232	19.010	9.637	2.496.211	1	33×	*Rothia dentocariosa* NCTC10917	96,17
D1P17	259.928	52.125	12.066	2.566.927	1	39×	*Rothia dentocariosa* NCTC10917	96,52
D3T4	2.206.376	23.696	7.854	2.296.981	1	226×	*Rothia mucilaginosa* ATCC2296	95,41
D4P7	263.877	114.000	8.649	2.511.012	1	42×	*Rothia dentocariosa* NCTC10917	96,31
D4T4	3.773.501	19.976	11.083	2.283.903	1	390×	*Rothia mucilaginosa* ATCC2296	93,87
D4T6	207.423	27.874	10.981	2.276.594	1	35×	*Rothia mucilaginosa* ATCC2296	93,87
D4T9	3.731.259	70.368	8.875	2.297.550	1	382×	*Rothia mucilaginosa* ATCC2296	93,87
D5T11	365.174	941	9.342	924.739*^2^	30	55×	*Rothia mucilaginosa* ATCC2296	94,54

* An ANI value >94% to the reference genome is considered to correspond to the same species (Richter and Rosselló-Móra, 2009). ^*2^In this case, the genome size could not be estimated and the sum of the contigs is presented.

Average nucleotide identity (ANI) values for the whole genomes confirmed that the five isolates from plaque corresponded to *R. dentocariosa* (D1P10, D1P17, D1P15, and D4P7) and *Rothia aeria* (D1P7), while all isolates from the tongue were closely related to the reference strain of *R. mucilaginosa* ATCC2296. Interestingly, when comparing the 10 genomes with each other, it appeared that isolates from the tongue of donor 4 (D4T4, D4T6, and D4T9) could belong to the same strain (sequence similarity at homologous regions > 99.9%, Table 2) and all *R. dentocariosa* studied were clearly different strains (ANI values 95–97%), indicating that within the same individual there may be substantial intra-specific genetic heterogeneity, confirming the phenotypic heterogeneity previously detected (Figure 2 and Table 1).

According to a FAO/WHO (2002), newly registered probiotic strains must be examined in pathological, genetic, toxicological, immunological, and microbiological aspects that could be relevant for human safety (FAO/WHO, 2002). Thus, mobile genetic elements (MGEs), antimicrobial resistance genes (ARGs) and virulence factors databases were used to determine if the 10 genomes contained any of these genetic elements, which could make them unsuitable for probiotic usage.

No virulence factors nor ARGs were found in any of the studied genomes (Supplementary Table S4). Several MGEs were found in the genomes of D1P7 and D1P17. These MGEs corresponded to methyltransferases or transposases previously identified in *Corynebacterium* species, which could correspond to horizontal gene transfer events from this genus, which is a common inhabitant of the oral cavity. Specifically, D1P7 had a double insertion of a transposase (tnp1249) in two close genomic regions and both D1P7 and D1P17 had three insertions of three different 23 rRNA methyltransferases: erm(X), ermCX, and ermLP. Based on the genome analysis all 10 isolate could be suitable strains for probiotics due to their absence of known virulence factors and antibiotic resistance genes.

**FIGURE 4:**
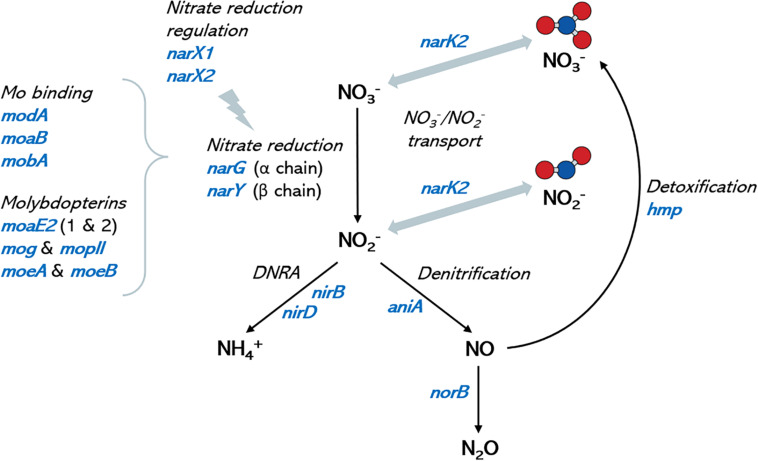
Nitrate metabolism genes in oral *Rothia* isolates. Graph shows a schematic representation of the nitrate metabolic pathways (depicted in black italic text) and the corresponding coding genes (in blue) that have been identified in the genomes of the oral isolates sequenced in the current manuscript. Molybdenum is a cofactor in nitrate reductase enzymes and identified molybdenum-related genes are included for reference. No genes for the reduction of nitrous oxide (N_2_O) to nitrogen (N_2_), nor for the oxidation of ammonia to nitrite were found. DNRA: dissimilatory nitrate reduction to ammonia. Ammonium production is proposed as a mechanism of pH buffering in the oral cavity, and nitric oxide is an antimicrobial molecule. A full list of genes in the different isolates can be found in Supplementary Data Sheet 1.

Genome annotation revealed that all selected isolates contained genes encoding nitrate transport proteins, nitrate to nitrite reduction, denitrification and DNRA (dissimilatory nitrate reduction to ammonia) enzymes (Figure 4). In addition, the genes encoding further reduction of nitric oxide to nitrous oxide (N_2_O) and detoxification of nitric oxide to nitrate (*hmp* gene) were also found, but no gene for reduction of N_2_O to nitrogen (N_2_) was detected, nor for fixation of N_2_ to ammonia. A large set of molybdenum (Mb) transport proteins and molybdopterins was also found, in agreement with molybdenum being a vital cofactor for bacterial nitrate reduction enzymes. A diverse array for genes involved in lactate metabolism was also present in all fully sequenced strains. A full list of genes involved in nitrate metabolic pathways and lactate utilization are shown in Supplementary Data Sheet 1.

### Isolate-Specific Effect of pH on Nitrate-Reduction

**FIGURE 5:**
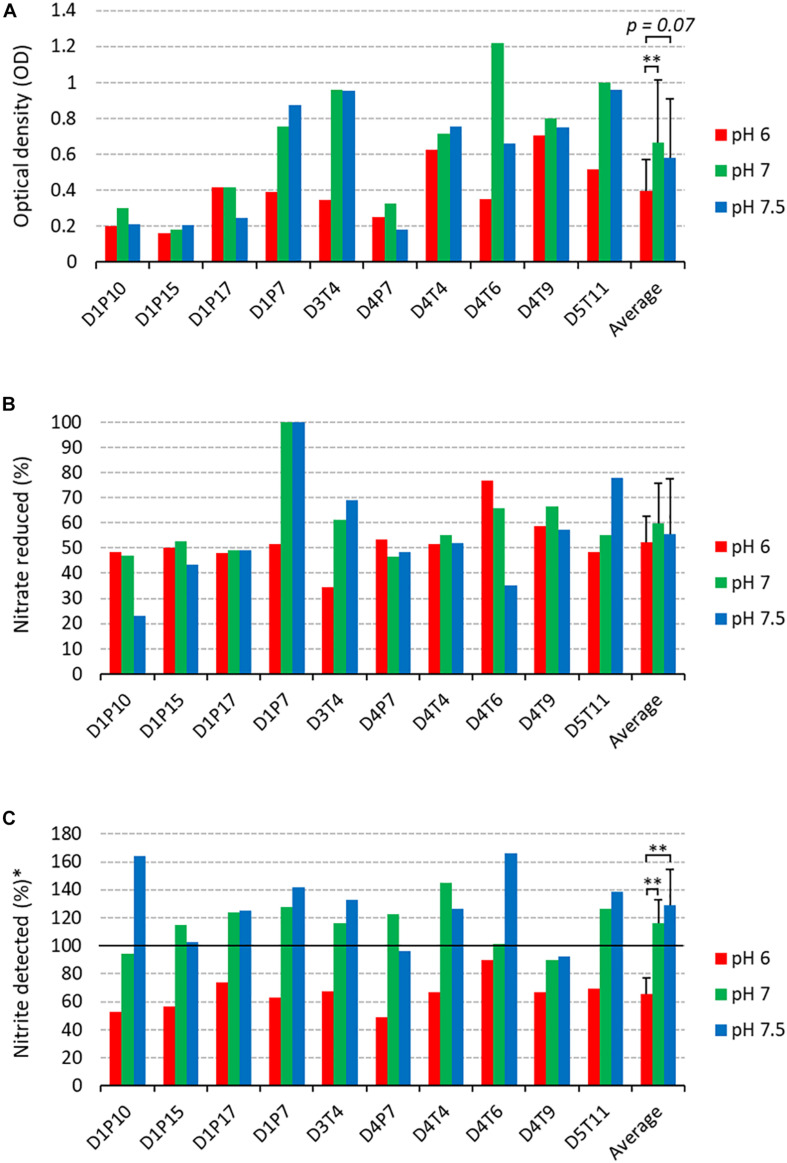
Nitrate reduction capacity of isolates at 3 pH levels (2nd screening). Bars show results after 5 h of incubation (37°C) at pH 6 (red bars), pH 7 (green bars), and pH 7.5 (blue bars) under aerobic conditions with starting OD 0.01. **(A)** Final optical density values. **(B)** Percentage of initially added nitrate (6.5 mM) that had been used up after 4 and 7 h of incubation. **(C)** Amount of nitrite detected, represented as a percentage of nitrate reduced (100 × mM nitrite detected/mM nitrate reduced). Reference codes of the 10 isolates are shown at the bottom, where D relates to the donor, T refers to tongue coating and P to dental plaque samples. ^∗∗^*p* < 0.01 according to a Wilcoxon test comparing all 10 isolates at different pH levels.

The 10 selected isolates were incubated during 5 h with 6.5 mM of nitrate at three different pH levels (pH 6.0, 7.0, and 7.5, Figure 5). There were some isolate-dependent effects of the pH on nitrate reduction. For example, D1P7 reduced 100% of nitrate after 5 h of incubation at pH 7.5 and pH 7, but reduced 51.72% of nitrate when grown at pH 6.0. Opposite to D1P7, D4T6 reduced 76.67% of nitrate when pH was 6.0, but it reduced only 35.19% of nitrate at a pH of 7.5.

The amount of nitrite detected was normalized by the amount of nitrate reduced, taking into account a 1:1 molar conversion of nitrate to nitrite (nitrite detected, Figure 5C). Interestingly, at pH 6.0 more nitrite appeared to be further reduced to other compounds compared to pH 7 and pH 7.5 (both *p* < 0.01). Specifically, 65.46% (*SD* 11.48%) of the reduced nitrate was detected as nitrite, which means that the other 34.54% had been converted to other compounds (e.g., reduction to nitric oxide or ammonia). It should be noted that no ammonium was detected in any of the cultures. At pH 7 and 7.5, the average percentage of nitrite detected was 116.16% (*SD* 16.76%) and 128.62% (*SD* 25.75%). This implies that 16.16% and 28.62%, respectively, of nitrite detected could not be explained by reduction of the 6.5 mM added nitrate, indicating that nitrite is, in part, being produced by other pathways than nitrate reduction.

Six isolates (D1P7, D3T4, D4T4, D4T6, D4T9, and D5T11) reduced nitrate equal to or above the median at two or three of the pH levels (Supplementary Table S5) and were selected to test their effects when added to *in vitro* complex oral communities.

### Effect of Isolates on *in vitro* Oral Community Metabolism

**FIGURE 6:**
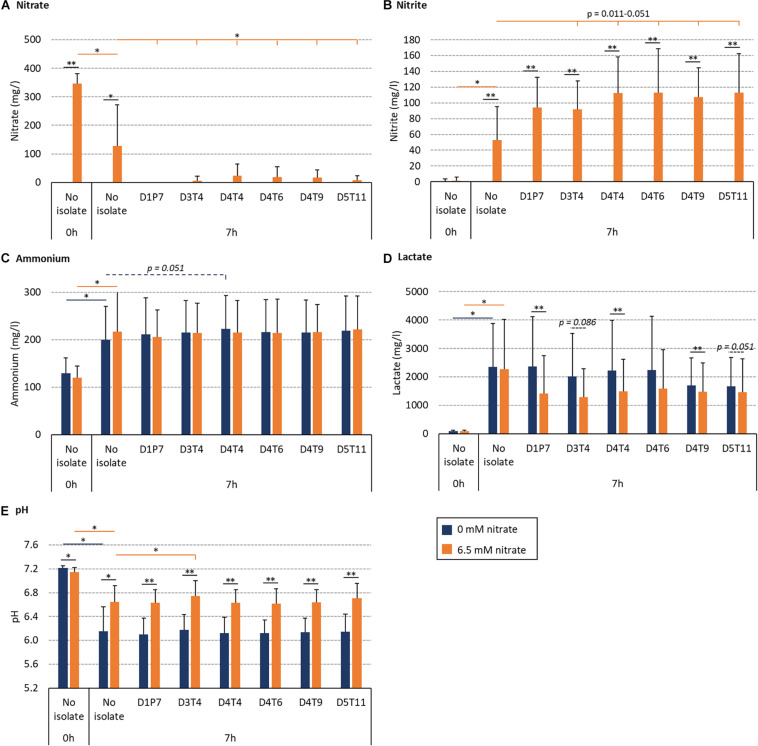
Effect of nitrate and six nitrate-reducing isolates on oral microcosm community metabolism. Bar plots show averages and standard deviations of supernatant measurements. Saliva of 9 donors was incubated with nutrient-rich medium to form *in vitro* oral biofilm with 6.5 mM nitrate (orange) or without nitrate (blue). Values correspond to measurements before incubation (0 h, which is the initial mixture of saliva and medium) and after 7 h of incubation. **(A)** Nitrate (mg/l), **(B)** nitrite (mg/l), **(C)** ammonium (mg/l), **(D)** lactate (mg/l), and **(E)** pH. All conditions with and without nitrate were compared (black lines with *p*-values). Additionally, conditions with isolates (D3T4, D4T4, D4T6, D4T9, and D5T11) were compared with the conditions without an isolate (i.e., no isolate): dark blue lines with *p*-values compare 0 mM nitrate conditions and orange lines with *p*-values compare 6.5 mM nitrate conditions. ^∗^*p* < 0.05, ^∗∗^*p* < 0.01 according to a Wilcoxon test.

The effect of the *R. aeria* isolate D1P7 and five *R. mucilaginosa* isolates (D3T4, D4T4, D4T6, D4T9, and D5T11) on oral community metabolism was tested *in vitro.* For this, biofilms were grown from saliva of nine different donors during 7 h with and without 6.5 mM nitrate (Figure 6). In these nine independent experiments, the levels of reduced nitrate were higher in the presence of any of the six isolates compared to the “no isolate” condition (*p* < 0.05, Figure 6A). Furthermore, the addition of isolates to the *in vitro* oral communities led to more nitrite production (Figure 6B), which was significant for D4T4, D4T6, D4T9, and D5T11 (*p* < 0.05), but not D1P7 (*p* = 0.11) and D3T4 (*p* = 0.051). Interestingly, D1P7 addition led to 100% of nitrate reduction in all donors, which indicates that nitrite was further metabolized into other compounds (e.g., ammonia or nitric oxide) when adding this isolate.

The BHI medium contains glucose (2 g/l) and this leads to acidification by oral communities over time. Regarding this, lactate correlated negatively with pH (*R* = −0.820, *p* < 0.01 in the control condition and −0.778, *p* < 0.05 in the nitrate condition). Importantly, 6.5 mM nitrate supplementation always led to a smaller decrease in pH after 7 h (Figure 6E) compared to 0 mM nitrate (*p* < 0.05). In communities with added isolates, this appeared to be, at least partly, due to lactate consumption: lower amounts of lactate were detected when combining nitrate with isolates compared to isolates without nitrate (Figure 6D, *p* = 0.008–0.110), which was significant for D1P7, D4T4, and D4T9 (*p* < 0.05), whereas there were no significant differences in the detected levels of ammonium. Only one isolate (D3T4), when combined with nitrate (i.e., symbiotic combination), significantly prevented the pH drop due to sugar metabolism more than when just adding nitrate without any of the isolates (i.e., a prebiotic treatment, *p* < 0.05). Another isolate, D4T4, showed a trend of increasing ammonium production without nitrate addition (i.e., a probiotic treatment, *p* = 0.051).

### Nitrate Reduction and Ammonium Production in Communities Without Isolates

**FIGURE 7:**
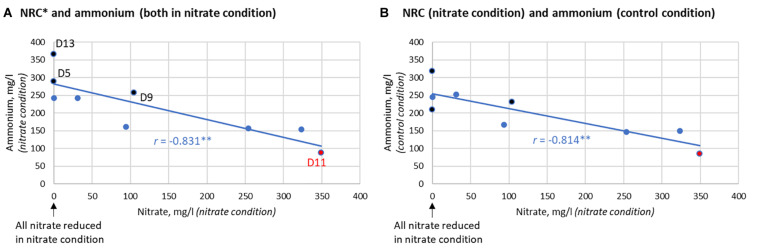
Correlation between nitrate reduction capacity (NRC) and ammonium production after 7 h. **(A)** Correlation between the nitrate reduction capacity (NRC), as determined by how much nitrate is left after incubation, and the ammonium detected in the nitrate condition (*R* = –0.831, *p* < 0.01). **(B)** Correlation between NRC and the ammonium produced in the control condition (*R* = –0.814, *p* < 0.01). This correlation indicates that the bacterial communities of donors that reduce nitrate efficiently are also capable of producing more ammonium in the absence of nitrate. The three donors that clearly produced more ammonium in the nitrate condition compared to the control condition are marked in black (from most to least extra ammonium production: D5 > D13 > D9). D5 and D13 were also two out of the three donors that reduced 100% of nitrate after 7 h. D11 had little to no nitrate reduction activity (marked in red).

In communities without isolates, there was no significant difference in lactate levels between the nitrate and control conditions after 7 h. Additionally, the ammonium detected did not differ significantly (*p* = 0.170). Nevertheless, the two individuals that produced the largest increase of ammonium resulting from nitrate supplementation (D5 and D13) reduced 100% of the nitrate after 7 h (Figure 7). Additionally, there was a clear correlation between the NRC of communities and ammonium production (*R* = −0.833, *p* < 0.01, between nitrate left and ammonium produced after 7 h, Figure 7A) which, unexpectedly, was also found in the control condition (*R* = −0.814, *p* < 0.01, Figure 7B). This indicates that communities with a better NRC are able to produce more ammonium even in the absence of nitrate, while nitrate can further increase ammonium accumulation in some individuals.

The DNA of the 7 h biofilms of two donors (D6 and D11, corresponding to a donor with above average and the lowest NRC, respectively) was analyzed by 16S rRNA Illumina sequencing. The isolates, which were added together with the saliva inoculum, appeared to colonize the initial biofilms successfully, as indicated by a higher relative abundance of the corresponding species in 16S rRNA sequencing data (Supplementary Table S6). The *Rothia* species increased more when adding the isolate and nitrate than when only adding the isolate (*p* < 0.05), which is an indication of active growth stimulated by nitrate.

### The Effect of Nitrate and Probiotic Addition in Donors With Different Nitrate Reduction Capacities (NRC)

When growing *in vitro* biofilms with the saliva of donor D11 in the presence of nitrate, nitrate did not decrease compared to the baseline (Figures 6, 7) and virtually no nitrite was produced, showing that the oral microbiota of this donor had a dramatically low NRC. However, a large percentage of nitrate was reduced when adding any of the isolates and the concentration of nitrite also increased notably (Figure 6B). This indicates that the addition of a probiotic was able to compensate the lack of NRC in this donor. When the same experiment was performed with the saliva of donors D5, D9, and D13, all added nitrate was reduced after 7 h, even in the absence of isolates. This suggests that these donors have a high NRC and the addition of nitrate alone is enough to promote nitrate reduction.

## Discussion

The nitrate–nitrite–nitric oxide pathway, which depends on nitrate-reduction by oral bacteria, contributes significantly to systemic nitric oxide levels and appears to be important for oral and systemic health (Doel et al., 2004; Kapil et al., 2013; Hezel and Weitzberg, 2015; Jockel-Schneider et al., 2016; Lundberg et al., 2018; Rosier et al., 2018; Rosier et al., 2020). Apart from the stimulation of this pathway with nitrate supplementation, which can result in beneficial cardio-metabolic and oral effects (Lundberg et al., 2018), nitrate reduction could also be promoted with the aid of nitrate-reducing probiotic bacteria (Rosier et al., 2018; Rosier et al., 2020). In the current *in vitro* study we isolate and select potential nitrate-reducing oral probiotics and demonstrate that the NRC of oral communities can be enhanced with newly isolated *Rothia* strains even in communities with little to no NRC. Our data show that nitrate by itself (prebiotic treatment) or combined with a nitrate-reducing isolate (symbiotic combination) prevented pH drops due to sugar metabolism, and some of the symbiotic combinations increased lactate consumption. A higher local pH and lactate consumption can prevent the development of cavities, suggesting a possible anti-caries potential of these isolates. Under certain conditions, nitrite was further reduced by isolates, but no ammonia was detected in the medium, suggesting that part of it may be converted to nitric oxide. Thus, future work should evaluate the potential contribution of some of these isolates to improve conditions that benefit from nitric oxide availability. This could be provided either by direct bacterial nitric oxide production or by production of nitrite and subsequent transformation into nitric oxide by the acidity of the stomach or at other body sites by human encoded pathways (Hezel and Weitzberg, 2015).

Some authors have criticized the use as oral probiotics of bacteria isolated from dairy products or from the gut, which may hamper their ability to colonize the oral cavity or provide undesired effects (Pham et al., 2009; López-López et al., 2017). We therefore isolated oral strains from healthy individuals and potentially probiotic candidates were selected based on their efficiency to perform an important function of the oral microbiota, namely nitrate reduction. Other selection criteria included a fast aerobic growth rate, lack of acidogenicity (a feature associated to tooth decay), ability to grow when added to *in vitro* oral communities and lack of virulence and antibiotic resistance genes. In total, 53 oxygen-tolerant nitrate-reducing bacteria were isolated from dental plaque or tongue coating samples from orally and systemically healthy individuals. In a first nitrate-reduction screening, 10/53 isolates were selected as potential probiotic candidates. In a second nitrate-reduction screening under three different pH levels, a final list of 6/10 candidates was completed. The effect of these six *Rothia* isolates on oral microbiota communities was studied, allowing us to evaluate their effect when added to a complex ecosystem, as a first step toward future animal or clinical studies.

### Effect on Oral Communities

The six *Rothia* isolates in the final selection were added to saliva and grown together in nutrient-rich medium for 7 h with or without 6.5 mM nitrate. In 7 h, initial biofilms grow with a highly similar bacterial composition to natural oral biofilms (Mira et al., 2019) and, together with planktonic cells, effect the pH and metabolite content of the supernatant. Metabolism of sugar in the medium leads to a decrease in pH, but both nitrate (prebiotic treatment) and the combination of any of the 6 isolates with nitrate (symbiotic combination) limited this decrease in pH compared to the control condition (*p* < 0.05). Li et al. (2007) added 1.5 mM nitrate to bacteria from saliva and also observed a limited sugar-derived pH drop. In a recent study, 6.5 mM nitrate under similar *in vitro* conditions as our study, prevented a pH drop after 5 and 9 h and this was shown to be due to ammonia production and lactate consumption (Rosier et al., 2020). In our current study, ammonia production and lactate consumption after nitrate supplementation was only observed in some cases, and therefore depended on the specific isolate under study or the donor’s saliva. Importantly, in both studies, a correlation was found between the NRC of oral communities and ammonia production with or without nitrate. This indicates that bacteria or bacterial processes involved in nitrate reduction are linked to ammonia production under these conditions.

The analysis of the genomes sequenced in the current manuscript reveals that all isolates also contain the gene encoding the nitrite reductase, which metabolizes nitrite into nitric oxide, and this gene is also present in other sequenced *Rothia* isolates. Some authors have determined that environmental factors like pH, the nitrogen:carbohydrate ratio (Kraft et al., 2014) and carbon source (Carlson et al., 2020) influence whether nitrite is converted to ammonia or to nitric oxide by aquatic or soil bacteria and future work should establish which factors switch the metabolic machinery of oral communities toward DNRA or denitrification. It is also notable that a wide array of genes involved in molybdenum transport and capture are present in the genomes of the oral *Rothia* isolates (Figure 4 and Supplementary Data Sheet 1). Thus, the strong molybdenum-related genomic content could partly explain the lower dental caries prevalence in areas of high molybdenum soil concentrations (Ludwig et al., 1960; Schroeder et al., 1970), as the dietary availability of this vital cofactor for nitrate reductase enzymes could contribute to the oral health benefits associated with nitrate reduction (Koopman et al., 2016).

An interesting finding in our study was that one isolate (D3T4), when combined with nitrate (i.e., symbiotic combination), significantly prevented the pH drop due to sugar metabolism more efficiently than when just adding nitrate without any of the isolates (i.e., a prebiotic treatment, *p* < 0.05). Another isolate, D4T4, showed a trend (*p* = 0.051) of increasing ammonium production without nitrate addition (i.e., a probiotic treatment). In relation to this, arginine is effectively used as a prebiotic to stimulate ammonia production and increase the local pH to prevent caries (Liu et al., 2012). The isolates D3T4 and D4T4 could therefore provide similar benefits and this should be further investigated *in vivo*. It should be noted that differences in ammonium detection can also be affected by ammonium consumption (an isolate may produce ammonia but metabolize it into other compounds or incorporate it as a nitrogen source). In fact, the detected levels of nitrite concentrations were often higher than the expected stoichiometric conversion of nitrate to nitrite (especially under alkaline pH), suggesting that nitrite could also be produced by other pathways (e.g., ammonia oxidation or nitrification). Our genome analysis, however, did not detect known ammonia oxidizing pathways (e.g., hydroxylamine oxidoreductase or ammonia monooxygenase) or the presence of genes for nitrogen fixation. Additionally, the oxidation of nitric oxide, which is a very unstable compound, could also favor accumulation of nitrite. Although our genome analysis did not detect bacterial nitric oxide synthases (NOS) that could convert arginine in nitric oxide, it did show the presence of flavohemoprotein, which detoxifies nitric oxide back to nitrate (Figure 4). Thus, future genomic and experimental work should aim to identify potential enzymes that could be producing nitrite or nitric oxide by other pathways in addition to nitrate reduction. In addition, future genetic and laboratory studies with controlled carbon, nitrogen and oxygen supplies should shed light on the complex nitrogen cycle that starts to be envisaged in oral communities, and more accurate techniques for measuring the highly unstable and dynamic nitrogen compounds, such as ozone chemiluminescence, may help in the characterization of all pathways involved.

Our data also show that, in the presence of nitrate, *Rothia* isolates can consume lactate, an organic acid which is produced by oral communities and associated to caries development (Bradshaw and Marsh, 1998), as less lactate was detected compared to the conditions of the isolates without nitrate (*p* = 0.008–0.110). On the one hand, nitrate has been shown to increase the salivary pH *in vivo* (Burleigh et al., 2019) and different *in vitro* studies, including this current work, show that nitrate provides resilience against acidification and lactate accumulation (Li et al., 2007; Rosier et al., 2020). On the other hand, nitrate-rich beetroot juice consumption has been shown to increase *Rothia* levels significantly compared to nitrate-depleted beetroot juice (Velmurugan et al., 2016; Vanhatalo et al., 2018). This indicates that *Rothia* species could contribute to the increase in pH and potentially also to resilience against acidification resulting from nitrate supplementation *in vivo*.

Importantly, many periodontitis-associated pathobionts are alkaliphiles and the effect of an increased salivary pH, which can result from nitrate supplementation (Burleigh et al., 2019), on oral communities *in vivo* should be investigated. Notably, previously, an increase in pH but a decrease in periodontitis-associated genera [some of which have been shown to be sensitive to nitric oxide (Backlund et al., 2014)] was observed after 5 h of incubation of oral communities with nitrate *in vitro* (Rosier et al., 2020). Additionally, nitrate-rich lettuce juice reduced gingival inflammation compared to nitrate-depleted lettuce juice in patients with chronic gingivitis (Jockel-Schneider et al., 2016). This provides preliminary evidence that nitrate supplementation could be beneficial for gum diseases, but this should be further investigated in clinical studies.

### Isolates’ Habitat Comparison and pH Preference

From all 53 nitrate-reducing isolates obtained in this study under aerobic conditions, 48 were *Rothia* species and five corresponded to *Actinomyces* species. Interestingly, all 23 *R. mucilaginosa* isolates originated from the tongue, while 20/21 *R. dentocariosa* isolates and all 4 *R. aeria* isolates originated from dental plaque. Recently, Wilbert et al. (2020) used Human Microbiome Project data analyzed by Eren et al. (2014) and concluded that *R. mucilaginosa* is strongly associated to the tongue (∼100-fold more abundant than on teeth), while *R. aeria* and *R. dentocariosa* appear to be strongly associated to teeth surfaces (>100-fold more abundant there than on the tongue). Our cultivation-based results confirm that *R. mucilaginosa* mostly lives on the tongue surface and *R. dentocariosa* and *R. aeria* on the teeth. Doel et al. (2005) also obtained 8 *Rothia* isolates from the tongue under aerobic conditions and most (6 out of 8) were *R. mucilaginosa*. In our study, after 4 h of incubation, *R. mucilaginosa* isolates had significantly grown to a higher optical density (*p* < 0.05) and reduced more nitrate than *R. dentocariosa* isolates (*p* < 0.01). Additionally, after 7 h, *R. mucilaginosa* isolates had reduced the pH 0.1 point more (*p* < 0.05), indicating possible differences in metabolism of these species.

The nitrate-reduction capacity of 10 probiotic candidates was evaluated at three different pH levels (pH 6, 7 and 7.5). Some isolates appeared to reduce nitrate at the same rate at all pH levels (e.g., D1P17 or D4T4), while others had a clear preference for a neutral and/or slightly alkaline pH (e.g., D1P7, D3T4, and D5T11) and one isolate reduced most nitrate at an acidic pH (D4T6). Regarding this, differences in oral pH levels among donors can result from host-specific factors such as salivary pH and dietary habits. Additionally, within a single donor, different habitats have different environmental conditions and pH gradients can be found within oral biofilms (Simón-Soro et al., 2013), which could explain intra-donor differences in the pH preference for nitrate-reduction of different stains.

Importantly, our data show that nitrite reduction was consistently stimulated by pH 6 compared to pH 7 and pH 7.5. Nitrite reduction of microbial communities can increase as the pH decreases (Cao et al., 2013). In the mouth this could have an important consequence for oral health, namely the prevention of acidification which is clearly associated with caries development. Arginine deiminase in oral probiotics has been shown to be activated by a low pH (López-López et al., 2017), leading to ammonia production and providing a self-regulatory feedback mechanism against acidification (Rosier et al., 2018). Likewise, nitrite reduction could be stimulated to increase the local pH by lactate consumption and ammonia production (DRNA) or antimicrobial nitric oxide release (denitrification). Similarly, lactate stimulates nitrite consumption which is performed in parallel with lactate consumption (Li et al., 2007). Importantly, non-enzymatic nitrite decomposition into nitric oxide occurs below pH 5.0 (Tiedje, 1988; Schreiber et al., 2010) and this was not the case of this experiment, indicating that enzymatic nitrite conversion of *Rothia* was stimulated by pH 6.0.

### The Associations With Health and Disease of *Rothia* and Nitrate

The clear association of *Rothia* with oral health has been discussed previously (Rosier et al., 2020). In short, *Rothia* species are found in higher relative abundance in oral biofilms when comparing healthy individuals with caries active, periodontitis or halitosis patients [see, for example, Belda-Ferre et al. (2012), Griffen et al. (2012), Meuric et al. (2017), Seerangaiyan et al. (2017)]. Related to this, *R. aeria* correlated negatively with inflammatory cytokines IL-17 and TNF-α in humans (Corrêa et al., 2019). Thus, multiple studies indicate that this is genus generally associated with oral health.

*Rothia* isolates have occasionally been isolated from, and associated with, endocarditis and other systemic disease samples (Binder et al., 1997), indicating that under certain conditions, there are strains that can translocate to other human niches. This is an extended feature of representatives of many oral species, which appear to be pre-adapted to attach to distant human tissues by their ability to adhere to oral mucosa components like collagen and fibronectin (García López and Martín-Galiano, 2020). As a consequence of that, many oral bacteria have been isolated from endocarditis samples, including classic commensals generally recognized as safe (“GRASS” organisms) such as *S. salivarius* (Corredoira et al., 2005), and different species of oral *Gemella*, *Granulicatella*, or *Prevotella*, among others (García López and Martín-Galiano, 2020). Likewise, probiotic Lactobacilli (also considered GRASS) have been isolated from the blood of immunosuppressed patients in intensive care units (Yelin et al., 2019). It is therefore crucial with all oral probiotics to select strains without virulence genes potentially involved in endocarditis or other diseases, which were shown to be absent in all 10 probiotic candidates selected in our study, and future work should perform animal trials to confirm their safety.

The safety and beneficial effects of dietary nitrate in general (Lundberg et al., 2018) and for the oral cavity (Rosier et al., 2020) were recently discussed. It should be noted that nitrate salts added to processed meat are associated with cancer (Bouvard et al., 2015; Etemadi et al., 2017). This nitrate is reduced to nitrite by bacteria in the meat and can further react with other molecules, such as heme, amines and amides, forming potentially carcinogenic *N*-nitroso compounds (Skibsted, 2011; Sindelar and Milkowski, 2012). However, we obtain most nitrate (>80%) from vegetables that are generally associated with health benefits and considered anticarcinogenic (Link and Potter, 2004; Wang et al., 2014; Turati et al., 2015). Antioxidants and polyphenols in fruits and vegetables prevent the formation, and possibly damage, of *N*-nitroso compounds (Chung et al., 2002; Ward, 2009; Kobayashi et al., 2015; Azqueta and Collins, 2016). In relation to this, different safety agencies concluded that epidemiological studies do not indicate that nitrate intake from diet or drinking water is associated with increased cancer risk (Chain, 2008). The concentrations of nitrate used in this study can be obtained in saliva by vegetable consumption. The application of topical doses of nitrate far below the acceptable daily intake (ADI, 3.7 mg/kg of body weight) would also be sufficient (Rosier et al., 2020). These could be obtained by oral products containing vegetable extracts or low amounts of nitrate salts in combination with antioxidants. The effect of nitrate-reducing probiotics and nitrate in combination with different dietary compounds (e.g., antioxidants and polyphenols) should be explored to make sure that potential future products do not result in harmful *N*-nitroso compounds formation.

### Experimental Conditions and Limitations

The aim of our study was to isolate aerobic fast-growing bacteria with a high NRC. All experiments were performed under aerobic conditions with BHI medium that contains 0.2% glucose as a carbon source. Doel et al. (2005) used a different medium (including tryptone, horse serum and 0.5% glucose) under aerobic and anaerobic conditions. In their study, most nitrite-producing isolates obtained in the presence of oxygen were *Rothia* (from the tongue) and *Actinomyces* (from saliva and plaque), but without oxygen, most nitrite-producing bacteria were *Veillonella* and *Actinomyces*, while no *Rothia* was detected. Other isolates in their study included *Staphylococcus*, *Corynebacterium*, and *Haemophilus*, all including species obtained with or without oxygen. The identification of these other species under aerobic conditions may have resulted from differences in medium composition or donor microbiota. Furthermore, in a recent study, it was shown that different carbon sources enrich different nitrate reducers of terrestrial environments (Carlson et al., 2020). Therefore, future work should focus on the isolation of a more diverse set of nitrate-reducing bacteria from the oral cavity using different growth conditions. Additionally, the effect of different nitrate-reducers and nitrate reduction rates on oral communities should be determined. In our study, we tested 6 *Rothia* isolates separately, but combining a variety of isolates, even from different species, could lead to mutualistic interactions and enhanced beneficial effects.

## Conclusion

Efficient nitrate reduction in the oral cavity has been clearly associated to human health but this metabolic pathway cannot be performed by humans, which lack the necessary enzymes. Thus, oral bacteria capable of nitrate reduction arise as a fascinating example of symbiosis by which the microbiome provides a health-associated benefit to the human host, which in exchange, recycles nitrate by actively concentrating plasma nitrate into the saliva (Hezel and Weitzberg, 2015). In fact, disruption of oral microbial communities by over-use of antiseptics or antibiotics will interfere with the nitrate–nitrite–nitric oxide pathway (Kapil et al., 2013), as well as food habits with low dietary nitrate (Ashworth et al., 2015). As a result of these, humans appear to vary widely in their NRC (Doel et al., 2004; Liddle et al., 2019), with important potential consequences for diseases or conditions that are influenced by a deficit in nitric oxide, ranging from cardiovascular diseases to reduced sport performance or diabetes development, among others (Lundberg et al., 2018). The current work shows that, in oral bacterial communities with a slightly reduced NRC, the supplementation of nitrate may suffice to restore and promote efficient nitrate reduction; however, our data also show that in individuals with extremely low NRC, the addition of a nitrate-reducing probiotic could be instrumental for a recovery of the function. Thus, future work should be performed to further characterize nitrate-reducing probiotics and test their potential efficacy in animal and clinical studies.

## Data Availability Statement

The original contributions presented in the study are publicly available. This data can be found in NCBI, under accession number PRJNA658327.

## Ethics Statement

The studies involving human participants were reviewed and approved by Ethical Committee of DGSP-FISABIO (Valencian Health Authority) with code 27-05-2016. The patients/participants provided their written informed consent to participate in this study.

## Author Contributions

BR and AM contributed to the design of the work and drafted and revised the manuscript. EM-G and BR did the experimental work. PC-E analyzed the bacterial genomes. BR, EM-G, and PC-E contributed to data acquisition and analysis. All authors read and approved the final manuscript.

## Conflict of Interest

AM and BR are co-inventors in a pending patent application owned by the FISABIO Institute, which protects different nitrate-reducing probiotics. The remaining authors declare that the research was conducted in the absence of any commercial or financial relationships that could be construed as a potential conflict of interest.

## Footnotes

1 http://expressionanalysis.github.io/ea-utils

## Supplementary Material

FIGURE S1 Comparison of two *Rothia* species from two donors. In the graph *R. mucilaginosa* and *R. dentocariosa* isolates of donor 1 and donor 4 are compared after 4 and 7 h of growth. Bars show the percentage of nitrate reduced **(A)**, optical density **(B),** and medium pH **(C)**. ^∗^*p* < 0.05, ^∗∗^*p* < 0.01 according to a Mann–Whitney *U* test.
