# Supplementary figures and images for: Isolation and Characterization of Nitrate-Reducing Bacteria as Potential Probiotics for Oral and Systemic Health
Source: Front Microbiol. 2020 Sep 15;11:555465. doi: 10.3389/fmicb.2020.555465 (PMC7522554; doi:10.3389/fmicb.2020.555465)

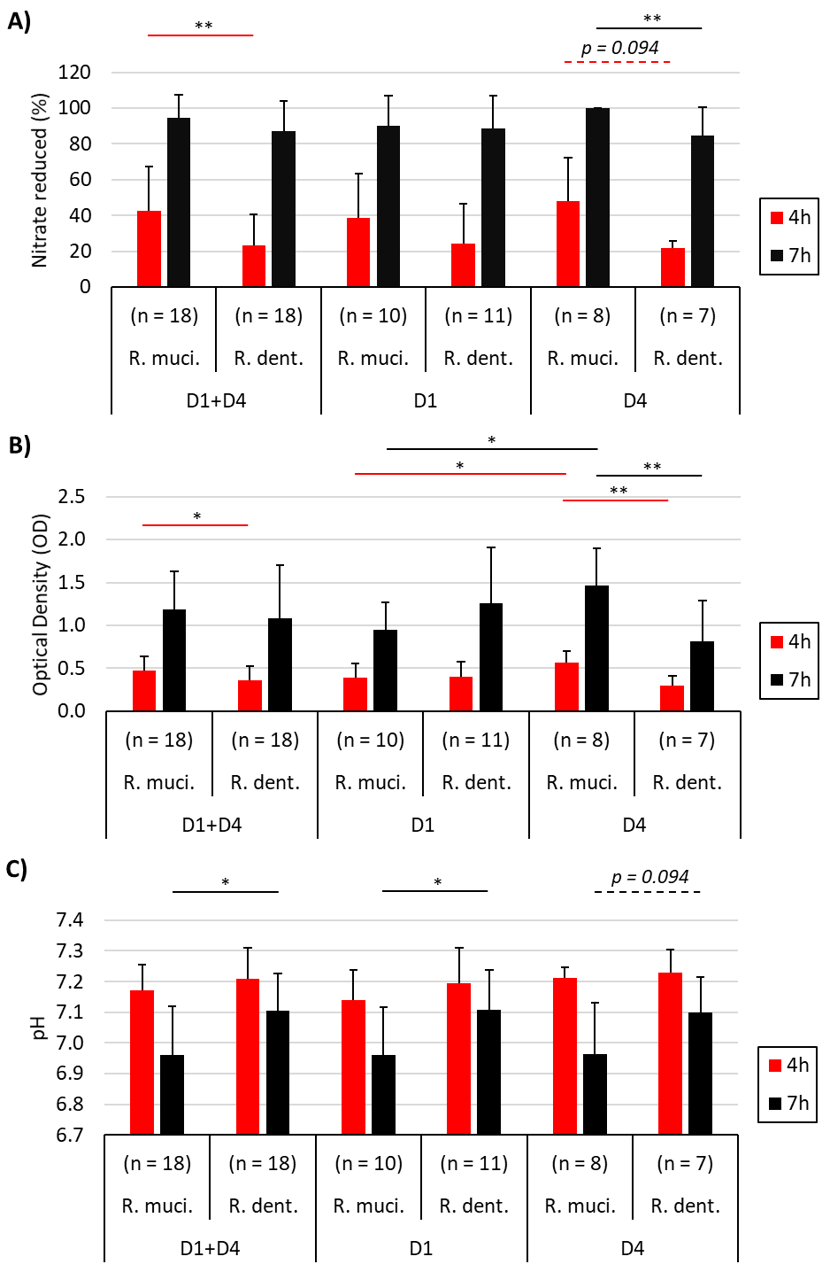

Supplement: FIGURE S1 — Comparison of two Rothia species from two donors. In the graph R. mucilaginosa and R. dentocariosa isolates of donor 1 and donor 4 are compared after 4 and 7 h of growth. Bars show the percentage of nitrate reduced (A), optical density (B), and medium pH (C). ∗p < 0.05, ∗∗p < 0.01 according to a Mann–Whitney U test. [file Image_1.TIF]
