# Supplementary material for: Isolation and Characterization of Nitrate-Reducing Bacteria as Potential Probiotics for Oral and Systemic Health
Source: Front Microbiol. 2020 Sep 15;11:555465. doi: 10.3389/fmicb.2020.555465 (PMC7522554; doi:10.3389/fmicb.2020.555465)
Supplement: Supplementary file 2 [file Data_Sheet_1.docx]

**Six supplementary tables (1-6)**

Supplementary Table 1: salivary properties and isolates from donors 1-5

|  | **Salivary pH*^1^** | **Salivary nitrite (mg/l)*^1^** | **Original sample** | | **Nitrite-producing isolates*^2^** | | **Confirmed nitrate-reducing isolates** | |  |
| --- | --- | --- | --- | --- | --- | --- | --- | --- | --- |
| **Donor 1** | 7.3 | 21 | Plaque | | 15 | | 15 | |  |
|  |  |  | Tongue | | 12 | | 10 | |  |
|  |  |  | Total | | 27 | | 25 | |  |
| **Donor 2** | 6.8 | 64 | Plaque | | 3 | | 3 | |  |
|  |  |  | Tongue | | 0 | | 0 | |  |
|  |  |  | Total | | 3 | | 3 | |  |
| **Donor 3** | 7.1 | 14 | Plaque | | 0 | | 0 | |  |
|  |  |  | Tongue | | 6 | | 4 | |  |
|  |  |  | Total | | 6 | | 4 | |  |
| **Donor 4** | 7.3 | 31 | Plaque | | 10 | | 9 | |  |
|  |  |  | Tongue | | 11 | | 9 | |  |
|  |  |  | Total | | 21 | | 18 | |  |
| **Donor 5** | 7.5 | 21 | Plaque | | 1 | | 1 | |  |
|  |  |  | Tongue | | 4 | | 2 | |  |
|  |  |  | Total | | 5 | | 3 | |  |
| **All (sum)** | | | | Plaque | | 29 | | 28 | |
|  |  |  |  | Tongue | | 33 | | 25 | |
|  |  |  |  | Total | | 62 | | 53 | |

*^1^Measured on a different day than sampling of plaque and tongue bacteria.

*^2^Colonies were selected that produced a red tone caused by a Griess reaction that stains nitrite

Supplementary Table 2: nitrite detected after 4h and 7h growth with 6.5 mM nitrate (continuation of table 1)

| **Isolate** | **Species (by 16S rRNA)** | **Nitrite detected (mM)** | |
| --- | --- | --- | --- |
|  |  | 4h | 7h |
| D1P10* | *Rothia dentocariosa* ATCC17931 | 4.00 | 7.09 |
| D3T4* | *Rothia mucilaginosa* DSM20746 | 5.43 | 1.32 |
| D1P7* | *Rothia aeria* A1-17B | 3.35 | 8.17 |
| D4T4* | *Rothia mucilaginosa* DSM20746 | 8.65 | 4.00 |
| D4T3 | *Rothia mucilaginosa* DSM20746 | 5.04 | 4.15 |
| D1P17* | *Rothia dentocariosa* ATCC17931 | 1.41 | 8.43 |
| D4T6* | *Rothia mucilaginosa* DSM20746 | 10.61 | 3.26 |
| D1P9 | *Rothia dentocariosa* ATCC17931 | 3.28 | 6.52 |
| D1P15* | *Rothia dentocariosa* ATCC17931 | 1.78 | 8.72 |
| D4T9* | *Rothia mucilaginosa* DSM20746 | 3.20 | 8.61 |
| D1P14 | *Rothia aeria* A1-17B | 2.93 | 7.13 |
| D5T11* | *Rothia mucilaginosa* DSM20746 | 4.64 | 9.22 |
| D1P12 | *Rothia aeria* A1-17B | 2.17 | 7.22 |
| D1T4 | *Rothia mucilaginosa* DSM20746 | 5.43 | 2.72 |
| D1T13 | *Rothia mucilaginosa* DSM20746 | 5.43 | 4.43 |
| D4T12 | *Rothia mucilaginosa* DSM20746 | 3.54 | 6.74 |
| D1P13 | *Rothia dentocariosa* ATCC17931 | 0.70 | 9.13 |
| D1T9 | *Rothia mucilaginosa* DSM20746 | 3.20 | 5.43 |
| D1T12 | *Rothia mucilaginosa* DSM20746 | 2.09 | 5.43 |
| D4T10 | *Rothia mucilaginosa* DSM20746 | 3.52 | 7.87 |
| D4T1A | *Rothia mucilaginosa* DSM20746 | 3.30 | 4.74 |
| D1T10 | *Rothia mucilaginosa* DSM20746 | 1.59 | 5.43 |
| D4T11 | *Rothia mucilaginosa* DSM20746 | 3.06 | 8.87 |
| D3T3 | *Rothia mucilaginosa* DSM20746 | 4.11 | 5.24 |
| D4P10 | *Rothia dentocariosa* ATCC17931 | 1.63 | 8.00 |
| D4P7* | *Rothia dentocariosa* ATCC17931 | 1.41 | 8.61 |
| D4P6 | *Rothia dentocariosa* ATCC17931 | 1.13 | 8.30 |
| D2P4 | *Rothia dentocariosa* ATCC17931 | 5.22 | 10.30 |
| D1T2 | *Rothia mucilaginosa* DSM20746 | 4.87 | 6.52 |
| D1P3 | *Actinomyces oris* ATCC27044 | 2.63 | 10.39 |
| D2P3 | *Rothia aeria* A1-17B | 6.48 | 7.69 |
| D1P5 | *Rothia dentocariosa* ATCC17931 | 6.13 | 7.04 |
| D1P4 | *Rothia dentocariosa* ATCC17931 | 6.56 | 7.30 |
| D1T11 | *Rothia mucilaginosa* DSM20746 | 0.89 | 5.43 |
| D4P1 | *Rothia dentocariosa* ATCC17931 | 1.59 | 6.96 |
| D1T6 | *Rothia mucilaginosa* DSM20746 | 2.89 | 5.43 |
| D1P16 | *Rothia dentocariosa* ATCC17931 | 0.54 | 7.13 |
| D1P6 | *Rothia dentocariosa* ATCC17931 | 0.74 | 7.09 |
| D2P2 | *Rothia dentocariosa* ATCC17931 | 2.35 | 7.69 |
| D1T3 | *Rothia mucilaginosa* DSM20746 | 2.61 | 5.43 |
| D4P11 | *Rothia dentocariosa* ATCC17931 | 0.70 | 6.17 |
| D4P9 | *Rothia dentocariosa* ATCC17931 | 0.98 | 4.83 |
| D5T8 | *Rothia mucilaginosa* DSM20746 | 1.48 | 7.22 |
| D4P8B | *Rothia dentocariosa* ATCC17931 | 0.93 | 4.02 |
| D3T1 | *Rothia dentocariosa* ATCC17931 | 1.02 | 4.87 |
| D4P3 | *Actinomyces viscosus* JCM8353 | 0.96 | 5.26 |
| D1P11 | *Rothia dentocariosa* ATCC17931 | 0.61 | 4.87 |
| D1P8 | *Rothia dentocariosa* ATCC17931 | 0.43 | 4.35 |
| D5P5 | *Actinomyces viscosus* JCM8353 | 1.04 | 3.91 |
| D1T1 | *Rothia mucilaginosa* DSM20746 | 2.02 | 5.17 |
| D4P4 | *Actinomyces viscosus* JCM8353 | 0.72 | 3.39 |
| D4T8 | *Actinomyces oris* JCM16131 | 0.91 | 1.20 |
| D3T2 | *Rothia mucilaginosa* DSM20746 | 0.50 | 1.76 |
| Average (*SD*) | - | 2.88  *(2.22)* | 6.15  *(2.17)* |

*selection of final 10 nitrate-reducing isolates of interest

Supplementary Table 3: Annotation results using Prokka v1.13.3 for the ten sequenced and

assembled genomes for this study.

| **Sample** | **Genes** | **Coding Regions** | **tRNA** | **rRNA** | **tmRNA** |
| --- | --- | --- | --- | --- | --- |
| **D1P7** | 2364 | 2304 | 49 | 8 | 1 |
| **D1P10** | 2190 | 2131 | 50 | 8 | 1 |
| **D1P15A** | 2194 | 2137 | 48 | 8 | 1 |
| **D1P17** | 2274 | 2216 | 49 | 8 | 1 |
| **D3T4** | 1879 | 1819 | 49 | 10 | 1 |
| **D4P7** | 2203 | 2144 | 50 | 8 | 1 |
| **D4T4** | 1878 | 1817 | 50 | 10 | 1 |
| **D4T6** | 1871 | 1811 | 49 | 10 | 1 |
| **D4T9** | 1899 | 1835 | 53 | 10 | 1 |
| **D5T11*** | 744 | 719 | 17 | 7 | 1 |

* Incomplete genome

Supplementary Table 4: Presence of possible Mobile Genetic Elements according to the ACLAME Database in the studied genomes of potential nitrate-reducing probiotics.

| **Sample** | **Gene Symbol** | **Gene Origin** | **Tax Name** | **Start** | **End** | **Identity (%)** | **Description** |
| --- | --- | --- | --- | --- | --- | --- | --- |
| D1P7 | erm(X) | plasmid | *Corynebacterium diphtheriae* | 1.586.366 | 1.587.390 | 99.90 | 23S rRNA methyltransferase |
| D1P7 | ermCX | plasmid | *Corynebacterium striatum* | 1.586.536 | 1.587.298 | 99.87 | 23S rRNA methyltransferase |
| D1P7 | ermLP | plasmid | *Corynebacterium striatum* | 1.586.389 | 1.586.436 | 100 | 23S rRNA methyltransferase |
| D1P7 | tnp1249 | plasmid | *Corynebacterium striatum* | 1.585.112 | 1.586.320 | 100 | Transposase |
| D1P7 | tnp1249 | plasmid | *Corynebacterium striatum* | 1.588.252 | 1.589.460 | 100 | Transposase |
| D1P17 | erm(X) | plasmid | *Corynebacterium diphtheriae* | 603.130 | 604.153 | 99.80 | 23S rRNA methyltransferase |
| D1P17 | ermCX | plasmid | *Corynebacterium striatum* | 603.299 | 604.061 | 99.87 | 23S rRNA methyltransferase |
| D1P17 | ermLP | plasmid | *Corynebacterium striatum* | 603.152 | 603.199 | 100 | 23S rRNA methyltransferase |

Supplementary table 5: nitrate reduced (%) by 10 selected isolated at 3 pH levels

|  | **pH 6** | **pH 7** | **pH 7.5** |
| --- | --- | --- | --- |
| **D1P10** | 48.28 | 46.88 | 23.08 |
| **D1P15** | 50.00 | 52.54 | 43.33 |
| **D1P17** | 48.15 | 49.15 | 49.02 |
| **D1P7** | **51.72*** | **100.00*** | **100.00*** |
| **D3T4** | 34.48 | **61.29*** | **68.97*** |
| **D4P7** | **53.33*** | 46.43 | 48.28 |
| **D4T4** | **51.72*** | **55.17*** | **52.00*** |
| **D4T6** | **76.67*** | **65.63*** | 35.19 |
| **D4T9** | **58.62*** | **66.67*** | **57.14*** |
| **D5T11** | 48.28 | **55.17*** | **77.78*** |
| *MEDIAN* | *50.86* | *55.17* | *50.51* |

*Isolate reduced a percentage equal to or higher than the median at this pH level

Supplementary table 6: Percentage of isolates in biofilms of two donors (D6 and D11) at 7h of growth

| Isolate | Condition | Donor 6 | | Donor 11 | |
| --- | --- | --- | --- | --- | --- |
|  |  | *R. mucilaginosa* (%) | *R. aeria* (%) | *R. mucilaginosa* (%) | *R. aeria* (%) |
| None | Control | 2.33 | 0.06 | 5.11 | 0.03 |
|  | Nitrate | 4.48 | 0.09 | 7.94 | 0.03 |
| D1P7  *R. aeria* | Control | 0.85 | 10.13 | 0.15 | 32.34 |
|  | Nitrate | 2.64 | 17.49 | 0.36 | 41.09 |
| D3T4  *R. mucilaginosa* | Control | 22.08 | 0.02 | 37.15 | 0.00 |
|  | Nitrate | 26.36 | 0.04 | 45.83 | 0.00 |
| D4T4  *R. mucilaginosa* | Control | 17.14 | 0.02 | 47.43 | 0.00 |
|  | Nitrate | 27.95 | 0.04 | 58.63 | 0.00 |
| D4T6  *R. mucilaginosa* | Control | 15.23 | 0.03 | 33.74 | 0.00 |
|  | Nitrate | 24.53 | 0.05 | 58.14 | 0.00 |
| D4T9  *R. mucilaginosa* | Control | 19.96 | 0.03 | 42.43 | 0.00 |
|  | Nitrate | 34.12 | 0.04 | 54.53 | 0.00 |
| D5T11A  *R. mucilaginosa* | Control | 5.14 | 0.04 | 35.15 | 0.00 |
|  | Nitrate | 14.43 | 0.04 | 40.72 | 0.00 |
